# The Role of* Rhodomyrtus tomentosa* (Aiton) Hassk. Fruits in Downregulation of Mast Cells-Mediated Allergic Responses

**DOI:** 10.1155/2019/3505034

**Published:** 2019-06-09

**Authors:** Thanh Sang Vo, Young-Sang Kim, Dai-Nghiep Ngo, Dai-Hung Ngo

**Affiliations:** ^1^NTT Hi-Tech Institute, Nguyen Tat Thanh University, Ho Chi Minh City, Vietnam; ^2^Department of Chemistry, Pukyong National University, Busan 608-737, Republic of Korea; ^3^Faculty of Biology and Biotechnology, University of Science, Vietnam National University, Ho Chi Minh City, Vietnam; ^4^Faculty of Natural Sciences, Thu Dau Mot University, Thu Dau Mot City, Binh Duong Province, Vietnam

## Abstract

*Rhodomyrtus tomentosa*, a flowering plant of Myrtaceae family from southern and southeastern Asia, was known to possess a rich source of structurally diverse and various biological activities. In this study, the inhibitory effect of* R. tomentosa* fruit extract (RFE) on allergic responses in calcium ionophore A23187-activated RBL-2H3 mast cells was investigated. The result showed that RFE was able to inhibit mast cell degranulation via decreasing *β*-hexosaminidase release and intracellular Ca^2+^ elevation at the concentration of 400 *μ*g/ml. Moreover, the suppressive effects of RFE on the production of interleukin-1*β* (IL-1*β*) and tumor necrosis factor-*α* (TNF-*α*) were evidenced. In addition, RFE effectively scavenged DPPH radical and suppressed the reactive oxygen species generation in a dose-dependent manner. Notably, the pretreatment of RFE caused the downregulation of tyrosine kinase Fyn phospholipid enzyme phospholipase C*γ* (PLC*γ*), extracellular-signal-regulated kinase (ERK), and nuclear factor kappa B (NF-*κ*B) phosphorylation. These results indicated that RFE could be a promising inhibitor of allergic responses and may be developed as bioactive ingredient for prevention or treatment of allergic diseases.

## 1. Introduction

Allergy is a hypersensitivity disorder that relates to exaggerated reaction of the immune system to harmless environmental substances such as animal dander, house dust mites, foods, pollen, insects, and chemical agents [1]. Allergic rhinitis, asthma, and atopic eczema are among the most common allergic diseases [2]. The prevalence, severity, and complexity of these diseases in the population are rapidly rising. So far, various medicines such as antihistamine, mast cell stabilizers, and immune suppressors have been applied for ameliorating allergic symptoms and reducing the suffering of anaphylaxis [3]. However, these medicines were not available to cure allergic diseases completely and exhibited several side effects [4]. Therefore, the discovery of safe and efficient therapeutics derived from natural products for prevention and treatment of allergic diseases is necessary.

*Rhodomyrtus tomentosa* is a flowering plant that belongs to the family Myrtaceae and is native to southern and southeastern Asia. It has been used in traditional Vietnamese, Chinese, and Malaysian medicine for a long time for treatment of diarrhea, dysentery, gynecopathy, stomachache, and wound-healing [5]. Moreover,* R. tomentosa* has been known to contain a rich source of structurally diverse and biologically active metabolites such as triterpenes, steroids, and phenolic compounds [6, 7]. In particular, various biological activities of* R. tomentosa* have been evaluated and reported recently [8]. Hence, it is considered as a potential source for exploring novel therapeutic agents. In the present study, the biological activity of* R. tomentosa* was further evaluated via investigating its inhibitory capacity on allergic responses* in vitro*.

Rat basophilic leukemia (RBL-2H3) cells display properties of mucosal-type mast cells. The activation of these cells leads to the release and generation of several inflammatory mediators [9]. Thus, RBL-2H3 cells have been commonly and successfully used in* in vitro* studies for screening antiallergic agents. Herein, RBL-2H3 cells were used as an* in vitro* model for evaluation of antiallergic activity of* R. tomentosa* fruit extract.

## 2. Materials and Methods

### 2.1. Materials

*R. tomentosa* fruits were purchased from Duong Dong Town, Phu Quoc district, Kien Giang province. ELISA kits were purchased from R&D Systems (Minneapolis, MN, USA). The antibodies were purchased from Santa Cruz Biotechnology Inc. (Santa Cruz, CA, USA). All other reagents were purchased from Sigma-Aldrich (St. Louis, MO, USA).

### 2.2. Extraction

*R. tomentosa* fruits were air-dried under shade and powdered using a grinder. The powder was soaked with ethanol 80% under the extract conditions of ratio (1/4, w/v), time (4 h), and temperature (60°C). The* R. tomentosa* fruit extract (RFE) was kept at 4°C for further investigation.

### 2.3. Cell Culture

RBL-2H3 cells (Korean Cell Line Bank, Seoul, Korea) were maintained in an incubator containing 5% CO_2_ at 37°C. The culture medium contains DMEM, 10% heat-inactivated FBS, 2 mM L-glutamine, 10 mM HEPES, 100 U/ml of penicillin G, and 100 mg/ml of streptomycin.

### 2.4. Cell Viability

The cytotoxic effect of the extract on RBL-2H3 cells was examined by MTT assay as previously described [10]. The cells (1x10^5^ cells/ml) were treated with the extract (100, 200, or 400 *μ*g/ml) for 24 h before incubation with MTT solution (1 mg/ml, final concentration) for 4 h. The supernatant was then removed, and DMSO (100 *μ*l) was added to solubilize the formed formazan salt. The absorbance was measured at 540 nm using a microplate reader (GENios® Tecan Austria GmbH, Austria). The cell viability was shown as a percentage compared to blank.

### 2.5. Degranulation Assay

RBL-2H3 cells (2 × 10^5^ cells/ml) were pretreated with the extract (100, 200, or 400 *μ*g/ml) for 24 h. The culture medium was replaced by Tyrode buffer before stimulation with calcium ionophore A23187 (1 *μ*M) for 30 min at 37°C. The level of *β*-hexosaminidase release was measured as previously described [11]. The *β*-hexosaminidase releases were calculated as a percentage compared to control: release ratio (%) = (T – B)/(C – B) × 100, where B is blank group, C is control group, and T is the tested group.

### 2.6. The Intracellular Ca^2+^ Elevation Assay

RBL-2H3 cells (5 × 10^4^ cells/ml) were pretreated with the extract (400 *μ*g/ml) for 24 h and subsequently incubated with Fura-3/AM (2 *μ*M) for 60 min at 37°C. The culture medium was then replaced by Tyrode buffer before exposure to calcium ionophore A23187 (1 *μ*M) for 5 min at 37°C. The Fura-3/AM fluorescence intensity was measured by a microplate reader (GENios Tecan, Austria, Grodigl/Salzburg, Austria) at 360 nm of excitation wavelength and 528 nm of emission wavelength [12].

### 2.7. Measurement of Cytokine Production

Different concentration of the extract (100, 200, or 400 *μ*g/ml) was added to the culture medium of RBL-2H3 cells (2 × 10^5^ cells/ml) for 24 h. The culture medium was then removed and Tyrode buffer was replaced before stimulation with calcium ionophore A23187 (1 *μ*M) for 1 h at 37°C. The supernatants were collected, and the amount of IL-1*β* or TNF-*α* was measured by ELISA kit.

### 2.8. 1,1-Diphenyl-2-Picryl-Hydrazyl Assay

The 1,1-diphenyl-2-picryl-hydrazyl (DPPH) assay was conducted as previously described [13]. Briefly, the mixture containing 100 *μ*l of the extract (400 *μ*g/ml) and 100 *μ*l of DPPH solution was incubated in the dark at room temperature for 30 min. The absorbance was then measured at 490 nm by Genova Nano (Jenway, UK). The DPPH radical scavenging ability was determined following the following formula:

(1) DPPH  scavenging  ability=ODcontrol−ODsampleODcontrol×100%

### 2.9. Measurement of Reactive Oxygen Species Production (ROS)

The extract (400 *μ*g/ml) was added to the culture medium of RBL-2H3 cells (1 × 10^3^ cells/ml) for 24 h before incubation with dihydroethidium (2 *μ*M) for 60 min at 37°C. The culture medium was then removed and Tyrode buffer was replaced before stimulation with calcium ionophore A23187 (1 *μ*M) for 30 min at 37°C. Paraformaldehyde 3% was used to fix the cells and the fluorescence intensity was conducted under a fluorescence microscope (CTR 6000, Leica, Wetzlar, Germany).

### 2.10. Western Blot Analysis

The protein expression level was measured by Western blot method. Various doses of the extract (100, 200, or 400 *μ*g/ml) were introduced to RBL-2H3 cells for 24 h prior to stimulation with calcium ionophore A23187 (1 *μ*M) for 30 min at 37°C. The procedure of protein detection was performed as previously described [12]. The protein band was visualized using LAS3000® Luminescent image analyzer (Fujifilm Life Science, Tokyo, Japan).

### 2.11. Statistical Analysis

Statistical analysis was performed by using the analysis of variance (ANOVA) test of Statistical Package for the Social Sciences (SPSS). The statistical significance of differences among groups was analyzed using Duncan's multiple range tests, wherein p < 0.05 was considered significant.

## 3. Results and Discussion

### 3.1. Effect of RFE on Mast Cell Degranulation

**Figure 1:**
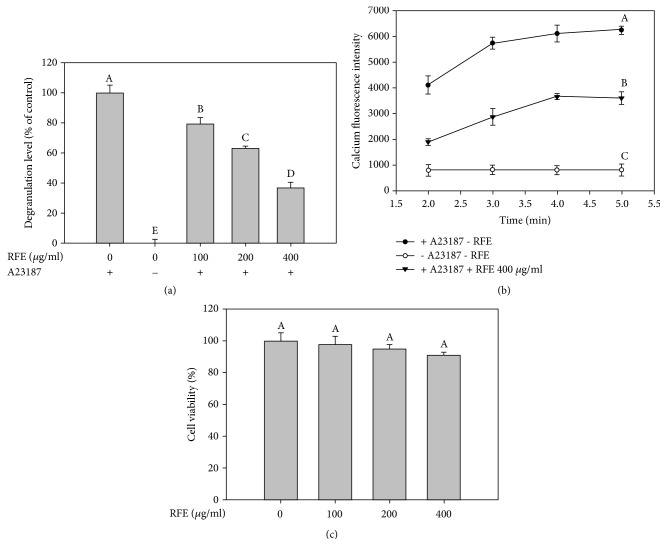
The inhibitory effect of RFE on mast cell degranulation in calcium ionophore A23187-activated RBL-2H3 cells ((a) and (b)) and cell viability (c). (a) The cells were treated with different concentrations of RFE for 24 h before stimulation with calcium ionophore A23187 for 30 min. The level of degranulation was measured via a spectrofluorometric assay. (b) The cells were pretreated with RFE for 24 h and incubated with Fura-3/AM for 1 h before exposure to calcium ionophore A23187 for 5 min. The Fura-3/AM fluorescence intensity was measured. (c) The cells were treated with various concentrations of RFE for 24 h. Cell viability was assessed by MTT method, and the results were expressed as percentage of surviving cells over blank cells (no addition of RFE). Each determination was made in three independent experiments, and the data are shown as means ± SD. Different letters A–E indicate significant difference among groups at* p* < 0.05 by Duncan's multiple-range test. The same letter indicates that the difference between the means is not statistically significant. If two groups have different letters, they are significantly different from each other at p < 0.05.

Mast cells play an important role in the development of allergic diseases and inflammatory processes [14]. Activation of mast cells triggers a cascade of intracellular events, especially degranulation [15]. Mast cell degranulation is considered to be one of the critical steps in allergic responses, causing the elevation of intracellular Ca^2+^ level and the subsequent release of various preformed mediators. These mediators are the origination of various pathophysiologic events in acute allergic responses [16]. Therefore, various antiallergic drugs have been developed so far, which are able to inhibit degranulation of mast cells. In this study, the inhibitory effect of RFE on mast degranulation was evaluated via measuring *β*-hexosaminidase release and intracellular Ca^2+^ elevation in the activated RBL-2H3 cells (Figure 1). It was observed that RFE was able to reduce *β*-hexosaminidase release to 79, 63, and 37% at the concentrations of 100, 200, and 400 *μ*g/ml (Figure 1(a)). Moreover, the increase in intracellular Ca^2+^ level induced by calcium ionophore A23187 was remarkably alleviated by RFE at concentration of 400 *μ*g/ml (Figure 1(b)). In particular, the inhibitory effect of RFE on mast cell degranulation was not due to cytotoxicity (Figure 1(c)). Notably, RFE possessed the similar inhibitory activity as compared with* Smilax glabra* [17],* Morinda citrifolia* [18], and* grapeseed extract* [19]. Evidently, calcium ionophore A23187 induces mast cell degranulation by increasing cell-membrane permeability [20, 21]. Thus, antiallergic agents having a membrane-stabilizing action may be desirable such as disodium cromoglycate or sodium hydroxypropylcromate. As a result, RFE may be suggested to stabilize the lipid bilayer membrane, thus reducing the degranulation in RBL-2H3 mast cells. Indeed, various phytochemical components of medicinal plants such as saponins, glycosides, flavonoids, tannins, and phenolic compounds have been evidenced as mast cell stabilizing agents [22]. Meanwhile, RFE has been reported to contain various tannins and phenolic compounds that may contribute to stabilizing mast cell membrane and preventing degranulation [23].

### 3.2. Effect of RFE on Cytokine Production

**Figure 2:**
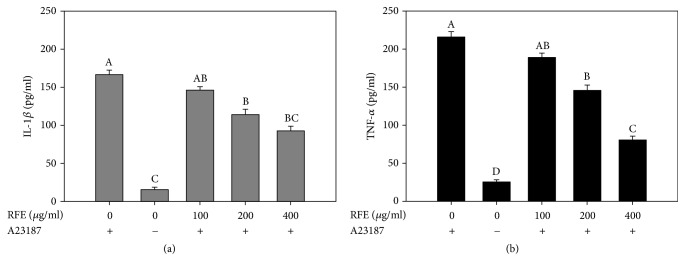
The suppressive effect of RFE on cytokine productions in calcium ionophore A23187-activated RBL-2H3 mast cells. Cells were pretreated with various concentrations of RFE for 24 h before exposure to A23187 for 1 h. The production level of IL-1*β* (a) and TNF-*α* (b) was quantified in culture media using commercial ELISA kits. Each determination was made in three independent experiments, and the data are shown as means ± SD. Different letters A–D indicate significant difference among groups (*p* < 0.05) by Duncan's multiple-range test. The same letter indicates that the difference between the means is not statistically significant. If two groups have different letters, they are significantly different from each other at p < 0.05. If a group is similar to A and B or B and C, it was addressed by letters “AB” or “BC,” respectively.

The allergic reactions are also characterized by the production of various cytokines. The activated mast cells are well established as an important source of several cytokines such as tumor necrosis factor-*α* (TNF-*α*), interleukin-1*β* (IL-1*β*), IL-4, IL-6, IL-8, and IL-13 [24]. The excessive production of these cytokines leads to the recruitment of inflammatory cells such as neutrophils and eosinophils, which increase the inflammatory responses [25]. Therefore, the decrease in cytokine production from the activated mast cells is one of the key indicators of the ameliorated allergic symptoms. Herein, the suppressive effect of RFE on IL-1*β* and TNF-*α* productions was investigated in RBL-2H3 mast cells. It was shown that the production levels of IL-1*β* and TNF-*α* were increased in the culture supernatants of A23187-exposed RBL-2H3 cells (Figure 2). The amount of IL-1*β* and TNF-*α* from the exposed cells was 167 ± 4.9 and 216 ± 6.4 pg/ml, respectively. Conversely, this increase was significantly reduced in a concentration-dependent manner by RFE pretreatment. At the concentration of 400 *μ*g/ml, RFE reduced IL-1*β* and TNF-*α* levels to 93 ± 5.7 and 80 ± 6 pg/ml, respectively.

### 3.3. Radical Scavenging Activity of RFE

**Figure 3:**
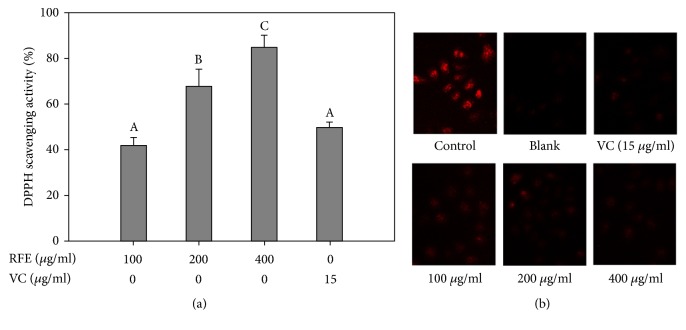
The radical scavenging activity of RFE. (a) 100 *μ*l of RFE (400 *μ*g/ml) was mixed with 100 *μ*l of DPPH solution for 30 min. The absorbance of the mixture was then measured at 490 nm. Each determination was made in three independent experiments, and the data are shown as means ± SD. Different letters A–C indicate significant difference among groups (*p* < 0.05) by Duncan's multiple-range test. If two groups have different letters, they are significantly different from each other at p < 0.05. (b) The cells were pretreated with RFE (400 *μ*g/ml) for 24 h and incubated with dihydroethidium for 1 h before exposure to calcium ionophore A23187 for 30 min. The level of ROS production was monitored by a light microscope with 10× magnification. Each determination was made in three independent experiments.

A free radical is considered as a molecule that contains one or more unpaired electrons in its outermost atomic or molecular orbital. It is generated from endogenous sources such as intracellular autooxidation and inactivation of small molecules or from exogenous sources such as tobacco smoke, certain pollutants, organic solvents, and pesticides [26]. The free radicals are recognized as agents involved in the pathogenesis of allergic diseases [27]. Meanwhile, the antioxidant agents from natural products have been proposed as an approach to reduce the allergic diseases [28]. Herein, RFE was determined to be effective in scavenging radicals (Figure 3). RFE was able to scavenge 85% DPPH radical at the concentration of 400 *μ*g/ml (Figure 3(a)). Moreover, the result from light microscope assay showed that the fluorescence density of ROS was markedly decreased in RFE-pretreated group as compared to A23187-stimulated group. It evidenced radicals as an inducer of Ca^2+^ elevation and subsequent degranulation in the mast cells [29]. Meanwhile, the blockade of ROS production by DPI or antioxidants such as (-)-epigallocatechin gallate suppressed mast cell degranulation [30, 31]. These results suggested that the antioxidant activity of RFE may contribute to the depression of mast cell degranulation.

### 3.4. Effect of RFE on the Intracellular Signaling Molecules in the Activated RBL-2H3 Mast Cells

**Figure 4:**
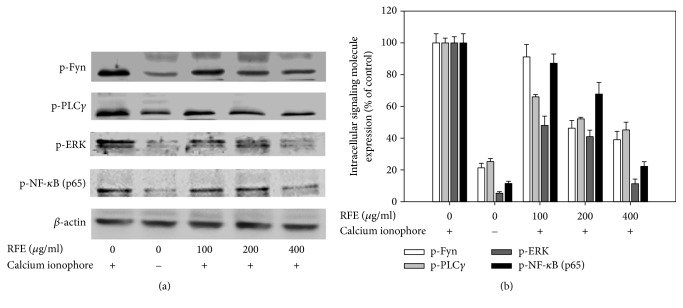
The downregulative effect of RFE on the intracellular signaling molecules in the activated RBL-2H3 mast cells. (a) The protein expression level was assessed by Western blotting. *β*-Actin was used as internal controls. Each determination was made in three independent experiments. (b) Densitometrically calculated expression levels of the intracellular signaling molecules were given as a percentage compared to the control group.

Evidently, the allergic responses were also indicated by the activation of a cascade of intracellular signaling molecules such as Fyn, PLC*γ*, MAPKs, and NF-*κ*B [15, 32]. It was reported that the activation of Fyn and PLC*γ* leads to microtubule polymerization, intracellular Ca^2+^ elevation, and subsequent mast cell degranulation [33, 34]. Meanwhile, the activation of NF-*κ*B triggers gene expression and production [32]. Thus, the inhibition of mast cell degranulation and cytokine production may relate to the inactivation of these intracellular signaling molecules. As shown in Figure 4, the phosphorylation of Fyn, PLC*γ*, ERK MAPK, and NF-*κ*B was increased in the control group exposed to A23187 alone. Conversely, the RFE pretreatment caused significant suppression on Fyn, PLC*γ*, ERK MAPK, and NF-*κ*B phosphorylation at the concentration of 400 *μ*g/ml. As a result, this suppressive effect of RFE may contribute to the inhibition of mast cell degranulation.

## 4. Conclusion

In conclusion, this study has evidenced the inhibitory effect of* R. tomentosa* fruit extract on allergic responses in A23187-activated RBL-2H3 mast cells. The inhibitory effect has been found due to decreasing *β*-hexosaminidase release and cytokine productions, suppressing ROS production, and downregulating the phosphorylation of tyrosine kinase Fyn and phospholipid enzyme phospholipase C*γ*. Therefore,* R. tomentosa* fruits could offer an attractive strategy for the control of allergic diseases. However, further studies related to safety and efficacy need to be evaluated.

## Data Availability

The data used to support the findings of this study are available from the corresponding author upon request.
